# Role of national psychiatric associations against pseudoscientific and misleading information and practices concerning mental health

**DOI:** 10.1192/j.eurpsy.2025.57

**Published:** 2025-08-26

**Authors:** K. Başar

**Affiliations:** Psychiatry, Hacettepe University, Ankara, Türkiye

## Abstract

**Abstract:**

Since their emergence as healthcare practices with scientific backgrounds, psychiatry and psychology have been criticized and attacked in different ways. Their scientific base has often been questioned, but more generally, their applications were suggested to follow alternative motives rather than the highest benefit of the suffering individuals. The antipsychiatry movement, with decades of history, on some occasions, could be considered to play an important role in optimizing psychiatric practices. However, with the increasing societal influence of antiscientific and pseudoscientific discourses, a significant section of individuals with mental health issues experience delays in their access to appropriate care and are sometimes harmed by the practices recommended as alternatives to psychiatry or medicine in general. There has been an increase in such misleading discourses following the pandemic, probably based on the uncertainty and lack of information on the nature of the infection and prevention methods, including the vaccine. In some instances, these misleading attitudes toward psychiatry and psychiatric practices are also pioneered by medical professionals. Some medical professionals deny the need and effectiveness of psychiatric practices, and some employ or recommend methods of care despite their lack of training and experience and the questionable nature of the practices. In many countries, national psychiatric associations are considered a leading source of information concerning mental health-related issues. However, without a systematic, need- and problem-focused strategy, including publicly available and acceptable information resources, it is difficult to oppose the overflow of misleading information currently available. Laws and regulations in many countries may act as a barrier against practices with no evidence base or with evidence of harmful effects. However, there are challenges in their implementation and limitations in their scope. National psychiatric associations may need to be involved in creating and implementing such regulations.

**Disclosure of Interest:**

None Declared

